# Facial Paralysis Treatment and Facial Symmetrization with Botulinum Neurotoxin: A Narrative Review with Illustrative Clinical Cases

**DOI:** 10.3390/toxins18060253

**Published:** 2026-06-04

**Authors:** Monica Renga, Roberta D’Emilio, Giovanni Salti, Selene Mogavero, Marco Papagni, Federico Biglioli, Alessandro Lozza

**Affiliations:** 1 Executive Committee of Agorà, Italian Society of Aesthetic Medicine, 20122 Milan, Italy; 2 Private Practice, 00139 Rome, Italy; 3 Medlight Institute, 50144 Florence, Italy; 4 Scientific Writing, 06089 Torgiano, Italy; 5 Maxillofacial Surgery Department, San Paolo Hospital, University of Milan, 20142 Milan, Italy; 6 Facial Paralysis Center, Studio Schiappadori, 20122 Milan, Italy; 7 CMP Medical Center, 27100 Pavia, Italy

**Keywords:** facial paralysis, botulinum neurotoxin, facial synkinesis, facial reanimation, chemodenervation, facial symmetry, neuromuscular retraining, electromyography-guided injections, ultrasound-guided injections, quality of life

## Abstract

Facial paralysis leads to static and dynamic asymmetry, hyperkinesis of the non-paralyzed side, and synkinesis, with major functional and psychosocial consequences for patients. Botulinum neurotoxin serotype A (BoNT-A) injections are an effective therapeutic option since they denervate overactive muscles, thereby reducing hyperkinesis or synkinesis and eventually improving asymmetry. This narrative review summarizes relevant literature on the use of BoNT-A for facial paralysis. It provides a summary of dosing strategies and treatment plans, discusses the use of functional scales for assessing facial paralysis and improvement after treatment, and outlines the use of electromyography (EMG) or ultrasound-guided injections to improve treatment outcomes. Finally, it discusses its potential role in the preparation for functional microsurgery. We also present the authors’ anecdotal experience, with three case reports: a woman with facial paralysis caused by Ramsay Hunt Syndrome, treated with a full-face and neck BoNT-A protocol and followed-up for 18 months with photographic documentation, to further assess the aesthetic improvement; a young woman with familiarity of facial paralysis, contralateral hyperkinesis, and synkinesis, managed with EMG-guided and landmark-guided injections; and her mother, with recurrent facial paralysis and chronic synkinesis, treated with stepwise BoNT-A sessions. All three cases demonstrated clinically meaningful improvements, as evidenced by the photographic material and functional grading scores presented.

## 1. Introduction

It is estimated that facial nerve disorders have an annual incidence of approximately 30/100,000 [1,2]. Bell’s palsy is the most common peripheral facial paralysis, accounting for 38–83% of cases. It has a lifetime prevalence of approximately 1.5%, with a recurrence rate of about 10% [3]. If left untreated, Bell’s palsy typically resolves in approximately 70% of cases. However, in the remaining 30% of cases, a partial recovery or aberrant reinnervation can occur, resulting in neurological symptoms. These include dysfunction of facial expression and motility, synkinesis, and hyperkinesis, which cause both functional and aesthetic damage [4,5].

Diagnosing the underlying cause and identifying the pattern of facial dysfunction are crucial for the management of facial palsy. This allows treatment to be tailored according to the onset, facial nerve injury, and the viability of the facial musculature.

Therapeutic strategies span medical therapy (including corticosteroids, antivirals, and antibiotics), corneal protection, physical therapy and neuromuscular retraining, chemodenervation agents such as botulinum neurotoxin serotype A (BoNT-A), fillers, and a range of static and dynamic surgical reanimation procedures [6,7,8,9,10].

### 1.1. Rationale for the Use of BoNT-A

Facial paralysis disrupts the delicate balance of mimetic muscles innervated by the facial nerve, leading to static and dynamic asymmetry, hyperkinesis of the non-paralyzed side, and synkinesis, with major functional and psychosocial consequences for patients [11,12]. BoNT-A provides a rational therapeutic option, as it produces a reversible chemical denervation of overactive muscles by blocking presynaptic acetylcholine release at the neuromuscular junction for 3–6 months. This allows selective weakening of hyperkinetic or synkinetic muscles while preserving the residual function of the paretic side [12,13,14]. In chronic facial paralysis and post-paralytic states, targeted BoNT-A injections into hyperactive muscles on the non-paralyzed side, for example, zygomaticus, levators, risorius, and depressor muscles, including the platysma (see [15] for a comprehensive anatomical study of the platysma muscle to improve facial and neck rejuvenation outcomes) and, when indicated, into synkinetic muscles on the affected side, can reduce contralateral hyperkinesis, improve resting and dynamic symmetry, and enhance the smile mechanism and overall facial appearance [11,12]. Clinical series and long-term experience show that such treatment is generally well tolerated, effectively decreases hyperkinesis and facial imbalance, and leads to high patient satisfaction, including better quality of life and emotional well-being [16]. Consequently, treatment with BoNT-A injections is considered a key intervention, which underscores its role as a key instrument in the long-term management of facial paralysis, often in combination with neuromuscular retraining or previous reconstructive procedures [10,12,17].

Within the “window of opportunity” during which BoNT-A interrupts abnormal neuromuscular transmission and thereby reduces simultaneous involuntary contractions, rehabilitation exercises that incorporate biofeedback mechanisms help patients train precise muscle contractions, leading to long-term improvement, even in chronic post-paretic synkinesis. Indeed, chemodenervation using BoNT-A is suggested to induce functional network reorganization in the brain, ultimately leading to motor recovery, beyond the pharmacological duration of the toxin [18,19,20].

Despite growing clinical experience with BoNT-A in facial paralysis, several important gaps and controversies remain. The evidence base consists predominantly of retrospective case series and small prospective cohorts, with only a limited number of randomized controlled trials [21,22]. Currently, there are no published official guidelines. Key unanswered questions include the optimal timing of BoNT-A initiation, the comparative benefits of landmark-guided versus instrumentally guided injections, and the role of formulation on effect duration.

### 1.2. Aim

This narrative review provides a critically appraised overview of BoNT-A treatment options currently adopted in clinical practice for the management of facial paralysis. Additionally, by presenting selected case reports from our clinical experience, this review offers practical elements that can assist practitioners in treating patients with facial paralysis using BoNT-A interventions. The clinical cases show facial muscle palsy with different origins and treatment times, classified as “early” or “late” relative to the onset of the palsy.

A literature search was performed using the following keywords: “facial OR face”, “paralysis OR palsy”, “botulinum neurotoxin OR BoNT OR botox OR botulin”, and combinations thereof. Articles were selected for their relevance to the main topic.

## 2. Clinical Cases

We now present three clinical cases selected to illustrate clinical scenarios in which BoNT-A may be applied: Case 1 demonstrates long-term management of chronic paralysis with full-face and neck treatment; Case 2 illustrates EMG- and landmark-guided injections in a young patient with familiarity for palsy and residual synkinesis; and Case 3 presents recurrent facial palsy with chronic synkinesis managed with a stepwise approach. Together, they cover a range of etiologies, disease durations, and treatment strategies and are presented as illustrative examples of individualized management.

Treatment parameters were individualized for each patient according to the distribution and severity of facial dysfunction, disease chronicity, and clinical response. The heterogeneity in doses, muscle targets, techniques, and intervals across the three cases reflects this individualization.

The treating clinicians performed functional assessments and were not blinded to treatment status; inter-rater reliability was not formally assessed, as this goes beyond the scope of this publication.

Written informed consent was obtained from all patients for the publication of their clinical data, photographs, and videos, and all procedures were conducted in accordance with the Declaration of Helsinki.

### 2.1. Case 1: Use of BoNT-A in a Patient with Facial Asymmetry of Neurological Origin

A 54-year-old female (born 1971) patient was referred to our clinic in 2024 for the treatment of facial asymmetry due to left peripheral facial nerve palsy. The patient developed Ramsay Hunt Syndrome in 2005. This caused the neurological injury, which led to dynamic alterations in facial movement, with concomitant issues of impaired hearing on the ipsilateral side. Early medications reported by the patient included antivirals, HD corticosteroids, Kabat physiotherapy, and acupuncture. In 2019, the patient received the first BoNT-A treatment of the right frontalis, with maintenance therapy every 6–8 months.

**Figure 1:**
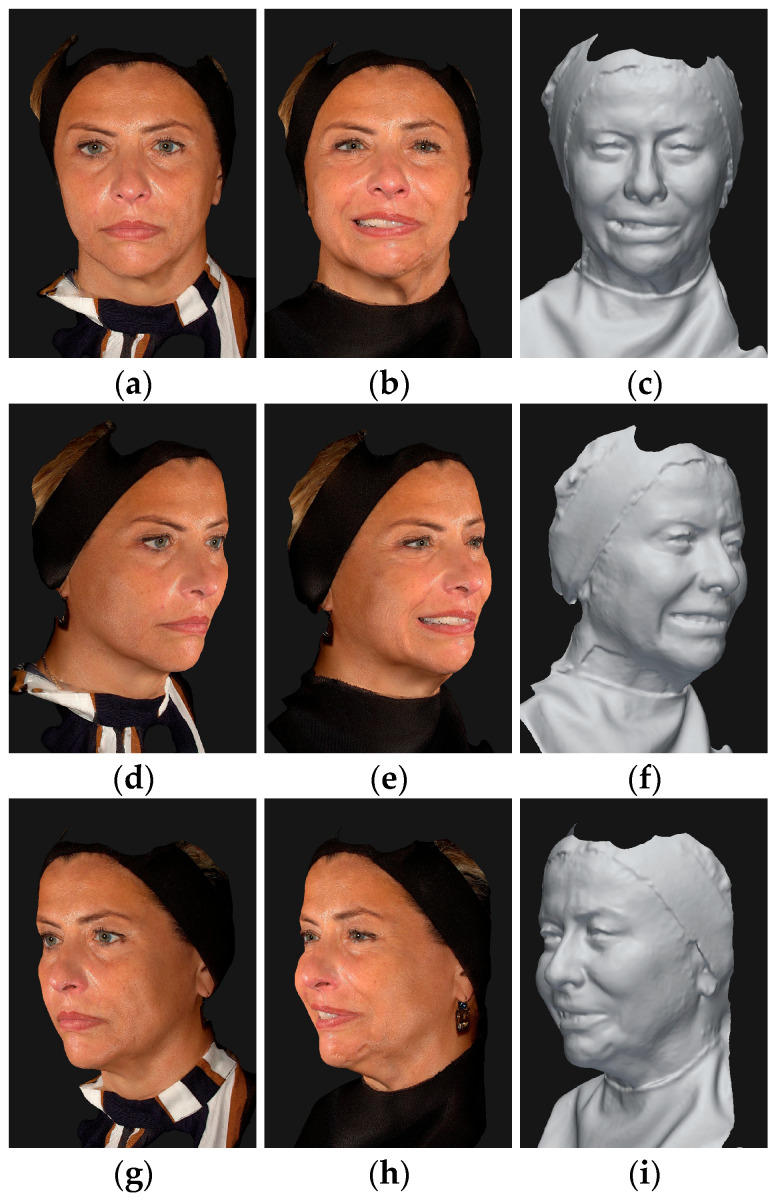
Facial appearance of a 54-year-old patient with left peripheral facial nerve palsy. Views: (**a**–**c**), front static–dynamic–gray-scale dynamic; (**d**–**f**), right side static–dynamic–gray-scale dynamic; (**g**–**i**), left side static–dynamic–gray-scale dynamic. Photographs were taken with the Vectra H2 3D imaging system^®^ (Canfield Scientific, Inc., Parsippany, NJ, USA).

During the first visit to our clinic, the patient exhibited hyperactivity of the muscles involved in smiling (both the elevator and depressor muscles) and of the platysma, whose hypertonicity contributed to a marked cervical prominence, leading to both psychological and aesthetic concerns. She refused to perform some of the facial expressions required for the functional and photographic assessment of the mimic muscles, as she was psychologically discouraged by her muscular impairment. Supplementary Video S1 (https://doi.org/10.5281/zenodo.19331961, accessed on 18 May 2026) shows the patient’s impairment when asked to produce a broad, open-mouth smile, with excessive exposure of the teeth and gingiva. Similarly, the images in Figure 1 show hyperactivity of the muscles of the middle and lower thirds on the unaffected side during dynamic movement, resulting in smile asymmetry with rightward deviation.

The muscles involved in the asymmetry were zygomaticus major, levator labii superioris (LLSAN), levator labii superioris (LLS), depressor anguli oris (DAO), depressor labii inferioris (DLI), and mentalis. Additionally, it is possible to observe concomitant hyperactivity of the platysma muscle on the affected side, particularly in its mandibular and labial portions, with the appearance of a platysmal band that alters the contour of the neck in both front and profile views. This is associated with hyperactivity of the mentalis muscle, resulting in multiple cutaneous irregularities in the chin region. Finally, a mild synkinesis of the muscles procerus, corrugator supercilia, depressor supercilia, and orbicularis oculi was observed during smiling on the affected side.

**Figure 2:**
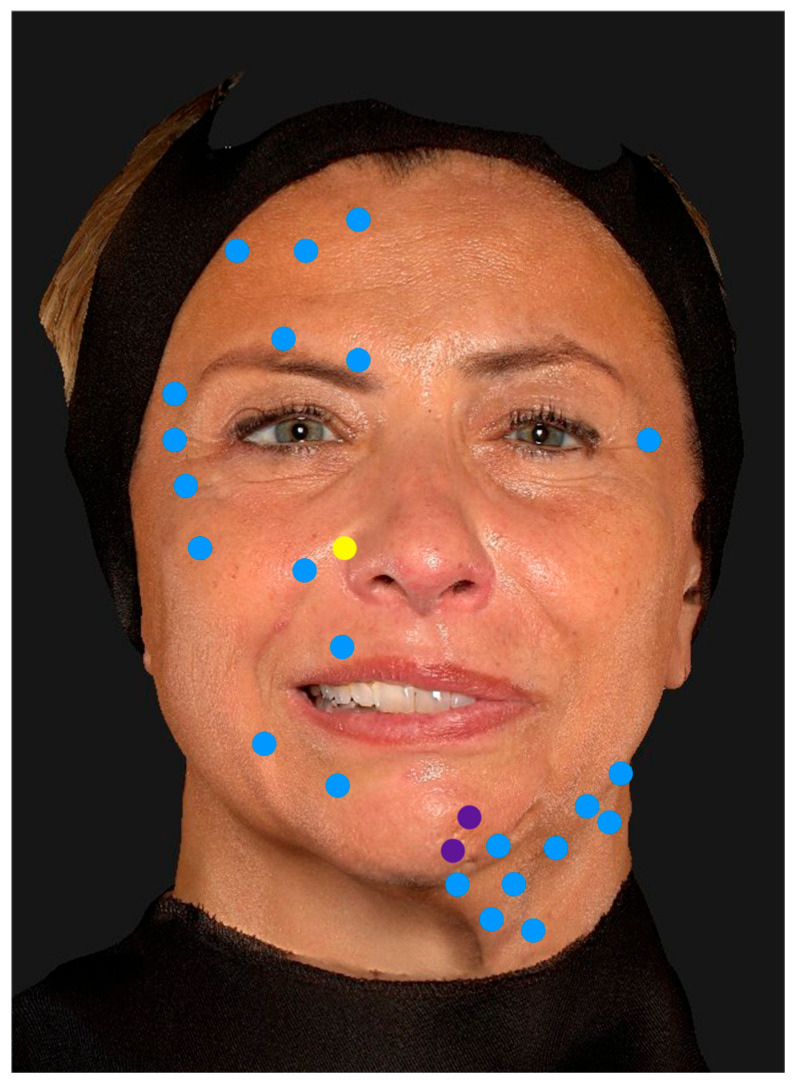
Injection sites and dosages for the first session. Yellow, 4 U; light blue, 2 U; purple, 1 U. Total injected: 52 U. The **right hemiface** was injected in the corrugator, frontalis, orbicularis, levator labii superioris alaeque nasi (LLSAN), zygomaticus major, levator labii superioris (LLS), depressor anguli oris (DAO), and depressor labii inferioris (DLI); the **left hemiface** was injected in the orbicularis, platysma (pars mandibularis, pars submandibularis, prominence), and mentalis. The photograph was taken with the Vectra H2 3D imaging system^®^.

For the first time, after nearly twenty years from the clinical onset of facial palsy, the patient received a complete BoNT-A treatment, addressed to all the functionally altered muscles of the face and neck. A total dose of 52 units (U) of onabotulinumtoxinA was injected, according to the scheme reported in Figure 2.

**Figure 3:**
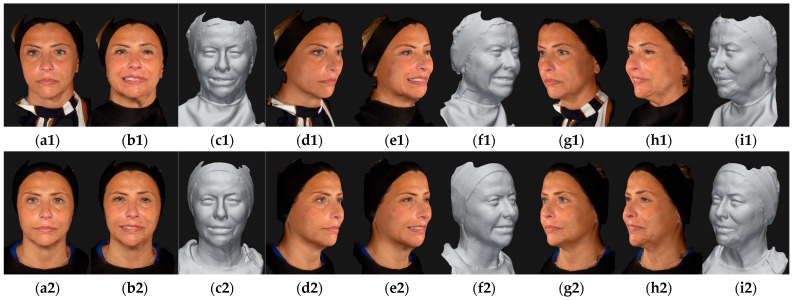

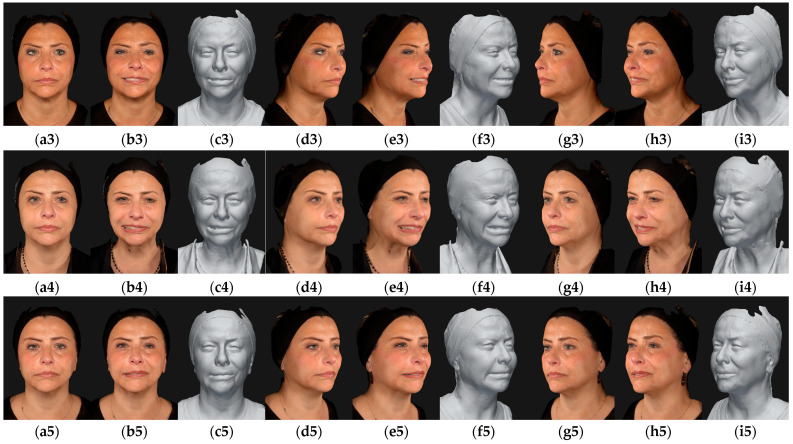
Facial appearance of a 54-year-old patient with left peripheral facial nerve palsy treated with onabotulinumtoxinA. Before the first treatment, September 2024 (**a1**–**i1**); before the second treatment, September 2025 (**a2**–**i2**); 2 weeks after the second treatment (**a3**–**i3**); before the third treatment, March 2026 (**a4**–**i4**); and 2 weeks after the third treatment (**a5**–**i5**). Views: (**a**–**c**), front static–dynamic–gray-scale dynamic; (**d**–**f**), right side static–dynamic–gray-scale dynamic; (**g**–**i**), left side static–dynamic–gray-scale dynamic. Photographs were taken with the Vectra H2 3D imaging system^®^.

We document how (three) consecutive treatments resulted in a progressive improvement in platysma prominence, producing a reshaping of the neck and a considerable overall aesthetic enhancement (Figure 3).

After three treatments, an evaluation during dynamic smiling shows a clear improvement in asymmetry. Neuromodulation of the platysma led to normalization of the neck, showing the disappearance of banding, the normalization of tightness, and of the submental contour and dimensions, along with resolution of the cutaneous “cobblestoning,” achieved through combined treatment of the mentalis and the mandibular portion of the platysma.

The patient’s Sunnybrook (SB) scores were 26 before the first treatment, 30 before the second treatment, 47 two weeks after the second treatment, 36 before the third treatment, and 48 two weeks after the third treatment (see Section 5: Use of functional scales). This reflects the early peak pharmacological effect of BoNT-A and shows a durable improvement even after its pharmacological effect has worn off.

### 2.2. Case 2: Use of BoNT-A in a Young Patient with Idiopathic Facial Palsy—The Daughter

A 25-year-old woman experienced facial palsy in February 2024. Notably, she had a familiarity with palsy, as her mother (Case 3, described later) also suffers from the same condition. This suggests a possible genetic susceptibility to facial nerve dysfunction.

Previous treatments included immediate therapy with nicety, antiviral, Vitamin B12, and cortisone. She continued with physiotherapy (Kabat method) and intravenous cortisone. The first improvements were noted in June 2024, but her condition then stabilized, with residual mild asymmetry at rest and hyperkinesia and synkinesis involving the DLI, mentalis, and platysma muscles on the right (contralateral) side, causing dynamic asymmetry and functional complaints during facial expression.

Her first approach to BoNT-A was in July 2025, when she underwent electromyography (EMG)-guided BoNT-A therapy.

Using EMG, the type of paresis and its outcomes were accurately characterized, with a detailed description of the extent of synkinesis, and the first procedure was performed under direct EMG guidance.

The patient presented with signs of aberrant reinnervation in the left facial nerve territory, resulting in synkinesis between the upper and lower facial regions. A left eye closure induced by activation of the perioral musculature was observed, along with muscular tension due to synkinetic activation of the left DAO and platysma during smiling.

OnabotulinumtoxinA was injected according to the following scheme, under EMG guidance: 9U into the left orbicularis oculi muscle (six injection points); 4.5 U into the left DAO; 18 U into the left platysma; 1.5 U into the left mentalis; 3 U into the right mentalis; 1.5 U into the right LLS; 3 U into the right DLI; and 1.5 U into the left buccinator, for a total dose of 42 U.

**Figure 4:**
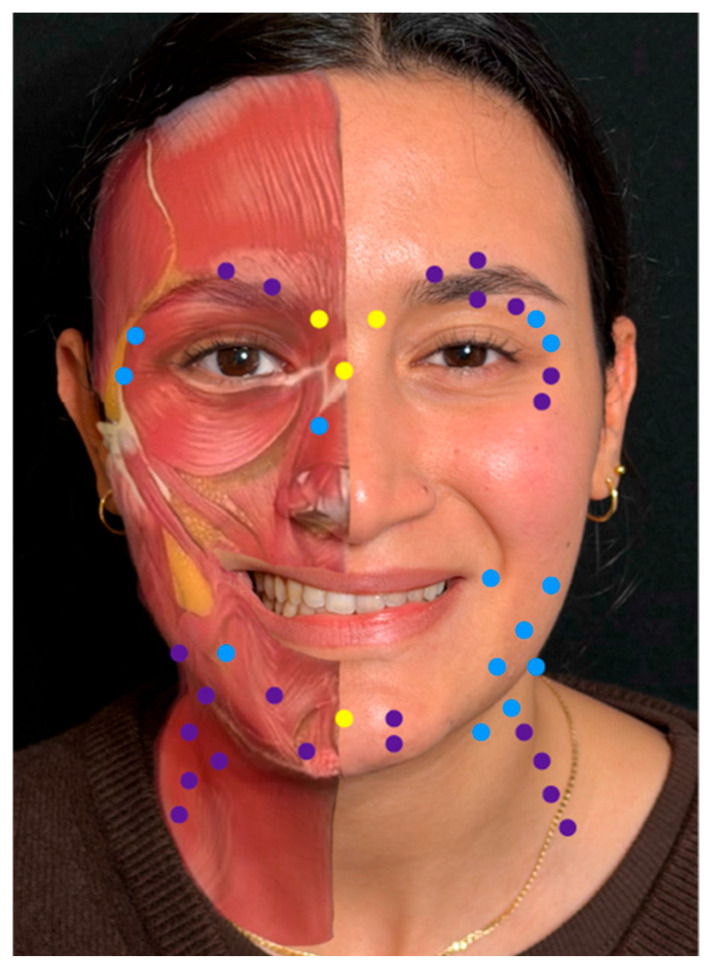
Points of injection and dosages for the second session, superimposed on a schematic of anatomical structures in a front static view. Yellow, 4 units (U) of onabotulinumtoxinA; light blue, 2 U; purple, 1 U. Total injected: 64 U.

In December 2025, she underwent her second session without EMG guidance. A total of 64 U of onabotulinumtoxinA was administered across multiple injection sites, with aliquots of 4, 2, and 1 U. Injection points are shown in Figure 4.

A control and retouch session was performed six weeks later, in January 2026: an additional total of 28 U of BoNT-A was injected in small aliquots of 2 U and 1 U to fine-tune asymmetries and residual synkinesis identified on clinical examination.

**Figure 5:**
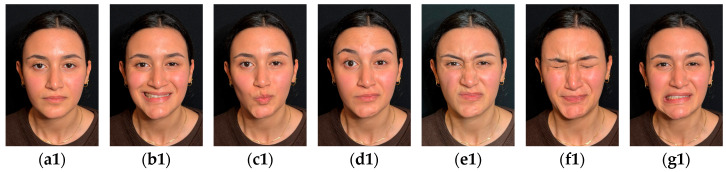

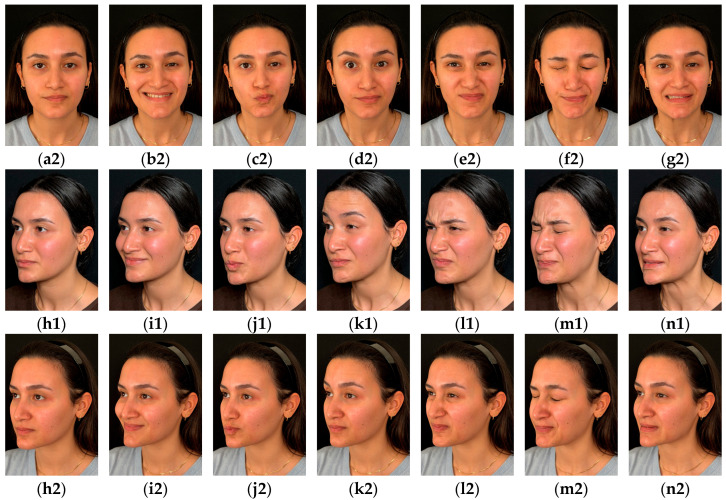
Facial appearance of a 25-year-old patient with left facial palsy treated with botulinum neurotoxin. Before treatment (**a1**–**n1**) and 6 weeks after treatment (**a2**–**n2**) views.

Clinical outcome was assessed through standardized photographic and video documentation across multiple facial expressions and views (Figure 5).

In the front and 45° static view, there was a clear improvement in global facial symmetry, with particular enhancement at the chin level (Figure 5(a1,a2,h1,h2)). In the smiling view, the patient demonstrated more symmetric smile dynamics with a reduction of hypercontractions of the DLI and mentalis muscles on the right side (Figure 5(b1,b2,i1,i2)).

During the kissing maneuver (Figure 5(c1,c2,j1,j2)), the front view showed improved fluidity of movements with reduced recruitment of the mentalis and a visible symmetrization of the face. In the front view during eyebrow elevation (Figure 5(d1,d2)), there was an improvement in the smoothness of the movement, with a significant reduction in involuntary movements in the lower third of the face. Symmetry at eye level also improved, with decreased spasm on the affected (left) side. The 45° view of the affected side during eyebrow elevation confirmed these findings (Figure 5(k1,k2)). In the expression of disgust, both in the front and 45° views (Figure 5(e1,e2,l1,l2)), there was a significant reduction in involuntary movements in the lower third of the face, with noticeable symmetrization of the upper lip and chin areas.

During forced eye closure (front and 45° views, Figure 5(f1,f2,m1,m2)), there was a significant reduction in involuntary movements in the mid and lower thirds of the face. Some residual descending synkinesis persisted and was addressed during the retouch session.

The evaluation during platysma contraction further highlighted the synergistic effect of treating the DLI, mentalis, and platysma muscles together. In the front view (Figure 5(g1,g2)), there was better symmetry of mouth movements with reduced hypercontractions of these three muscles, particularly on the right side. In the 45° view (Figure 5(n1,n2)) of the affected side during platysma activation, the patient showed more symmetric mouth movement, more balanced lower-lip repositioning, and a redefined, smoother chin profile.

The patient’s improvement on the SB scale was from 51 before treatment to 64, six weeks after treatment (see Section 5. Use of functional scales). This reflects the early peak pharmacological effect of BoNT-A. The durability of this improvement beyond the 3–6-month pharmacological window cannot be determined from the current follow-up data.

### 2.3. Case 3: Use of BoNT-A in a Patient with Recurrent Idiopathic Facial Palsy—The Mother

This is the case of a 51-year-old woman (mother of the previous patient, Case 2) who had her first right facial palsy manifestation at the age of 12 (in 1987). A second episode occurred in 2014, with a third episode in 2018. The recurrent episodes of facial palsy led to hyperkinesis and synkinesis, causing facial asymmetry at rest and during movement, as well as neck and facial tension.

The most affected muscles were the platysma and mentalis on the affected side.

**Figure 6:**
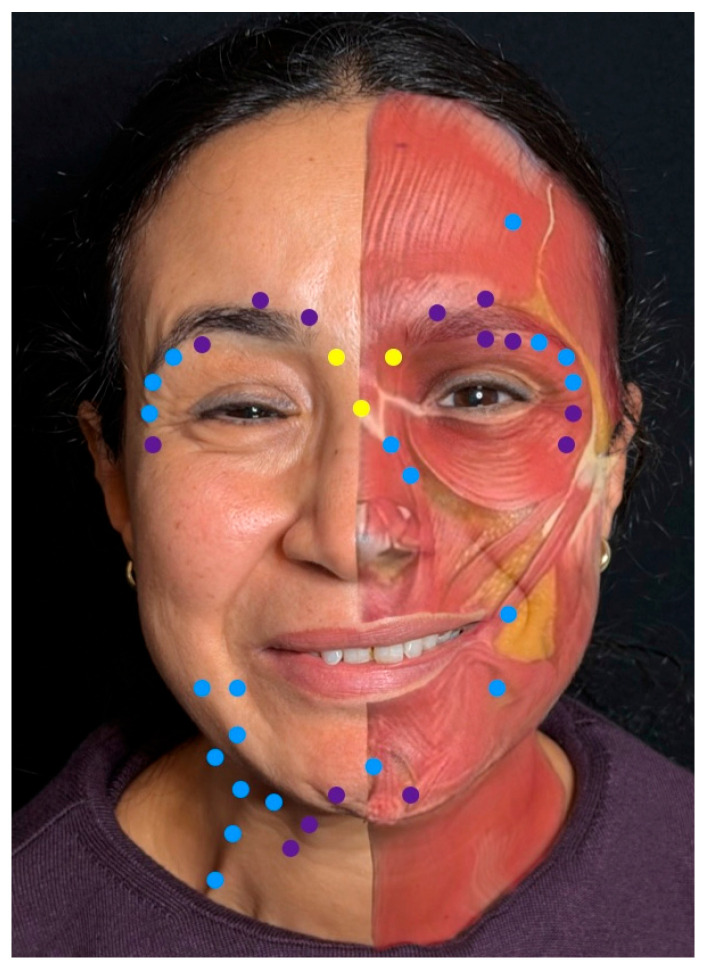
Points of injection and dosages superimposed on a schematic of anatomical structures in a front static view. Yellow, 4 units (U); light blue, 2 U; purple, 1 U. Total injected: 66 U.

BoNT-A treatment was administered in December 2025. A total of 66 U was injected at multiple injection sites, with doses of 4, 2, and 1 U (Figure 6).

A control and retouch session was performed 6 weeks later, in January 2026. Residual asymmetries and synkinetic movements were identified and targeted with additional injections. A total of 22 U of BoNT-A was injected in small doses of 2 U and 1 U.

**Figure 7:**
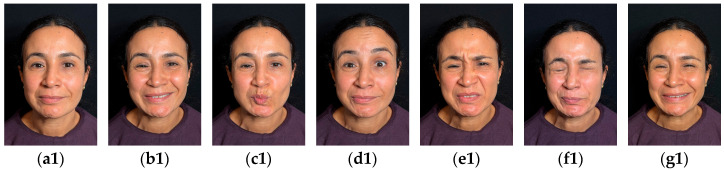

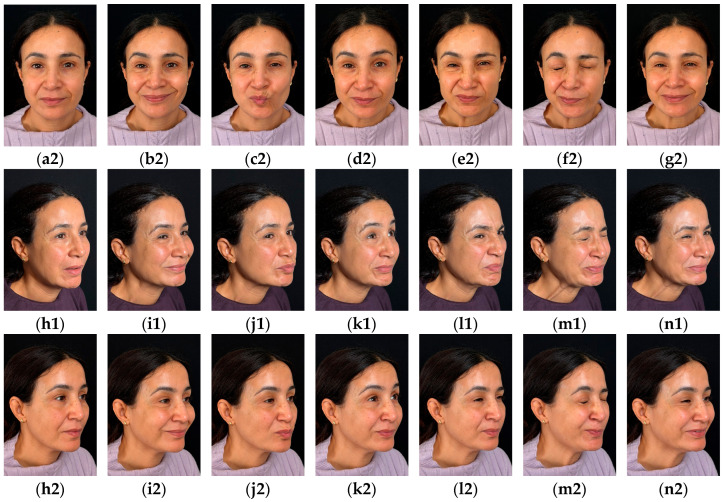
Facial appearance of a 51-year-old patient with left facial palsy treated with botulinum neurotoxin. Before treatment (**a1**–**n1**) and 6 weeks after treatment (**a2**–**n2**) views.

Clinical results were evaluated using standardized front and 45° angle photographic and video assessments across a range of facial expressions (Figure 7).

In the front and 45° static view, there was a clear improvement in global facial symmetry, with the complete disappearance of muscular hypertonus at the chin level (Figure 7(a1,a2,h1,h2)). In the front smiling view, smile dynamics were more symmetric, with reduced hypercontractions of the right eye, mentalis, and platysma. The face appeared not only more relaxed but also younger (Figure 7(b1,b2,i1,i2)).

During the kissing maneuver view, there was improved movement fluidity, reduced recruitment of the mentalis muscle, and notable facial symmetrization. Concomitantly, a reduction in platysma contraction was observed, accompanied by an improvement in the patient’s facial and neck tension (Figure 7(c1,c2,j1,j2)). The front and 45° view of the affected right side during eyebrow elevation showed smoother, more isolated brow lifting with a significant reduction in involuntary movements in the lower third of the face. Symmetry at eye level improved, with decreased spasm of the right eye (Figure 7(d1,d2,k1,k2)).

Front and 45° views during the expression of disgust showed a significant reduction in involuntary movements in the lower third of the face and visible symmetrization of both the upper lip and chin areas (Figure 7(e1,e2,l1,l2)).

During forced eye closure (front view), there was a significant reduction in involuntary movements in the middle and lower thirds of the face and in hypercontractions of the platysma muscle. Some residual descendant synkinesis was observed and corrected during the control visit 6 weeks later (Figure 7(f1,f2)). The 45° view shows the reduction in the intensity of descending synkinesis (Figure 7(m1,m2)).

Finally, during platysma contraction in the front and 45° views, the post-treatment face no longer exhibited the pronounced hypercontractions that had generated asymmetry and tension in the lower third and neck. The chin lost its hypercontraction, resulting in a more natural, defined profile (Figure 7(g1,g2,n1,n2)).

The patient’s improvement on the SB scale was from 47 before treatment to 58, six weeks after treatment (see Section 5. Use of functional scales). This reflects the early peak pharmacological effect of BoNT-A. The durability of this improvement beyond the 3–6-month pharmacological window cannot be determined from the current follow-up data.

No significant adverse events were recorded for our patients. Specifically, no iatrogenic asymmetry, muscle over-weakening, lagophthalmos, ptosis, oral incompetence, or dysphonia was observed. It should be noted, however, that selection bias exists because the presented clinical cases are only representative of the procedure’s protocol and outcomes.

## 3. Use of BoNT-A in Acute and Chronic Facial Paralysis

The classical distinction between acute and chronic facial paralysis relies on an 18- to 24-months-since-onset cut-off, which traditionally guides management. Recently, EMG has been used as a more precise tool for differentiating patients based on the presence of muscle fibrillations (i.e., viable muscles) that characterize acute disease [23].

The use of BoNT-A in acute facial paralysis is primarily useful for facial symmetrization, as treating the non-paralyzed side reduces asymmetry, thereby lessening the aesthetic impact and social interaction difficulties experienced by the patient. Furthermore, recent rehabilitation studies have shown that treatment of the healthy side can improve and accelerate the motor recovery of the paralyzed side by reducing overactivity in healthy muscles, thereby facilitating facial rebalancing, decreasing inhibitory feedback, and supporting more effective neuromuscular retraining [24,25,26].

In patients with chronic facial paralysis, some areas may become active in an erroneous manner, distorting facial expression and causing functional difficulties in both the upper and lower face due to incorrect reinnervation. This can lead to bothersome neurological symptoms, such as painful muscle spasms, and a distorted facial appearance in both static and dynamic conditions.

In general, BoNT-A is a minimally invasive option for addressing synkinesis on the affected side and hyperkinesis on the contralateral side, thus improving facial symmetry at rest and during movement. Three randomized trials, with a total of 105 patients, were identified in a systematic review in which BoNT-A improved objective measures, such as the vertical palpebral distance (VP), corneal light reflex to upper lid margin distance (MCRD), and clinical score for facial palsy, across different chronic cohorts [14]. The included studies also reported improvements in patients’ quality of life (QoL), including better social interaction, self-image, and peripheral visual function, as well as reduced perceived severity; patient satisfaction often persisted beyond the expected 3–6-month pharmacological effect. When combined with neuromuscular retraining or other physical therapies, BoNT-A appears to create a therapeutic “window of opportunity” during which relatively normal movement patterns can be trained, resulting in sustained reductions in synkinesis beyond the toxin’s duration of action.

The use of BoNT-A in facial paralysis is not exempt from adverse events. The most clinically significant risks include iatrogenic asymmetry resulting from relative over-weakening of the treated muscles, which may temporarily worsen the patient’s appearance and is most likely with excessive initial doses. For example, perioral muscle weakening can cause transient difficulties with drinking, eating, articulation, or puckering, especially after injections into the orbicularis oris, orbicularis oculi, and platysma muscles [27]. Adverse effects are mostly self-limiting and typically resolve within the toxin’s pharmacological effect window. This is an advantage of BoNT-A over surgery. However, they may cause significant patient distress and temporary functional impairment during this period, and patients should be advised accordingly before treatment. The systematic review by Cooper et al. noted that safety considerations should be taken into account, for example, after injections to treat oro-ocular synkinesis, which may worsen blepharoptosis, lagophthalmos, and diplopia [14]. Also, overtreatment can cause cosmetic and expression alterations (smile dysfunction or brow ptosis) or even loss of function, underscoring the need for refined dosing protocols.

Overall, the effects of BoNT-A treatment in patients with chronic facial paralysis have been documented in several other studies.

De Maio and Bento have injected a fixed dose of BoNT-A to treat contralateral hyperkinesis in 18 patients with unilateral facial palsy that emerged more than 1 year before treatment. The key injected muscles were zygomaticus major/minor, LLS, and LLSAN, risorius, modiolus region, DAO, and DLI. This approach uniformly weakened the oral and palpebral sphincters, achieving better symmetry. The degree of improvement was measured using a digital caliper (distance between the cheilion and the endocanthion, exocanthion, or tragion), indicating a significant reduction in muscle activity, with peaks of maximal improvement between 28 and 42 days post-treatment and a clinically relevant effect lasting up to 150–180 days. All patients were satisfied or very satisfied with the treatment, and all adverse events were mild, mostly characterized by early difficulties with drinking, chewing, speaking, or puckering, as patients adapted to the new perioral weakness [27].

From an aesthetic point of view, however, our Case 1 shows that the platysma region improves not only long-term but also over time and across multiple treatment sessions, with a concomitant volumetric reduction of the hyperactive muscle.

However, BoNT-A treatment can also improve drinking ability. In two retrospective case reports of patients with drooling and dependence on a straw, BoNT-A treatment on the contralateral side of the VIIth nerve palsy (in the buccinator, zygomaticus major, and orbicularis oris) decreased hyperkinesis in the healthy hemiface. This approach reduced the elevation of the mouth corner during smiling, allowing more symmetric lip closure and puckering, and enabling liquids to be held more evenly. The benefits lasted up to 3 months before a new treatment was necessary [28].

In a retrospective case-note review of 14 patients with long-standing facial palsy, the injection of BoNT-A with personalized doses into the contralateral smile muscles consistently improved active and passive movements. The effect began rapidly (on average, 6 days) and lasted 11 weeks; all patients reported improved facial symmetry. Interestingly, in this study, EMG was adopted to show muscle activity reduction after the injections [29]. This provides an objective measure of the treatment’s efficacy. The use of EMG to guide the injections will be discussed in a later paragraph of this review.

BoNT-A has also been used to improve facial symmetry in patients with chronic unilateral facial paralysis who had previously undergone reconstructive surgery [30]. The dosing strategy was individualized and targeted specific upper- and lower-face muscles on the non-paralyzed hemiface. The documented improvement consisted of a mean reduction in asymmetry (as measured by a clinical score) of 48.8% at 1 month post-treatment, with a residual improvement of 16.8% at 6 months. Notably, the late gain in symmetry reflected an 18% increase in the clinical score of the paralyzed side, suggesting that the temporary weakening of contralateral hyperactivity can, in turn, improve the functionality of reanimated or transplanted muscles and is associated with meaningful improvements in disease-specific quality of life [30].

Notably, the choice of BoNT-A formulation may influence the duration of the benefits. In a single-masked randomized clinical trial, 3 different BoNT-A formulations (onabotulinumtoxinA, abobotulinumtoxinA, and incobotulinumtoxinA) were administered to 28 patients, for a total of 38 treatment sessions. The Synkinesis Assessment Questionnaire (SAQ) showed that the three different formulations had similar early improvements (1–2 weeks post-treatment). However, the formulations diverged at 4 weeks: there was less improvement with incobotulinumtoxinA (17%) than with onabotulinumtoxinA (41%) or abobotulinumtoxinA (42%). These data suggest that although all three neuromodulators are effective in the short term, agents with longer durations, such as onabotulinumtoxinA and abobotulinumtoxinA, may provide more sustained control of synkinesis at standard dosing. In contrast, incobotulinumtoxinA may require higher doses or shorter intervals between sessions to achieve an equivalent clinical impact [21].

Taken together, the reviewed studies show positive outcomes with BoNT-A in facial paralysis. Still, they are limited by heterogeneous study designs, small sample sizes, inconsistent outcome measures, variable patient populations, short follow-up durations, and the incomplete reporting of adverse events. Large, well-powered randomized controlled trials with validated outcome measures and long-term follow-up remain the most significant unmet need in this field.

## 4. Treatment Plan

The collaboration among specialists facilitates the exchange of information required for the optimal treatment of facial deformities in the post-paralysis phase, with a focus on both functional and aesthetic outcomes. Treatment plans with low initial doses and, if necessary, incremental doses on the contralateral side of the face, to improve both functional and aesthetic balance, can be useful [31].

It should be noted that no internationally validated, evidence-based clinical practice guidelines currently exist for the use of BoNT-A in facial paralysis.

In one of our previous publications, we proposed that asymmetry and functional deficits, such as synkinesis, hyperkinesis, and age-related tissue descent, should first be documented using standardized photography in multiple positions and facial expressions, both static and dynamic, at each visit. We also recorded patient-reported outcomes using the Global Aesthetic Improvement Scale (GAIS) at each treatment session. Overall, the injection protocol relied on standardized entry points, dose ranges, and injection depths. The target muscles were not limited to those responsible for synkinesis, hyperkinesis, and facial asymmetry; they also included those that would benefit from BoNT-A treatment for aesthetic purposes. This maximized the overall patient satisfaction. In particular, target muscles for the affected side included the procerus, corrugator supercilia, frontalis, orbicularis oculi (including endopalpebral portion), nasalis (transverse portion), zygomaticus major, risorius, LLSAN, DLI, platysma (lateral and anterior), and mentalis. For the unaffected side, included muscles were the corrugator supercilii, frontalis, orbicularis oculi (including endopalpebral portion), nasalis (transverse portion), platysma (lateral and anterior), and mentalis [31].

In particular, eye closure from orbicularis muscle action and smile distortion, as well as downward deviation of the corner of the mouth from hyperactivity of the platysma and DAO muscles, may be the first targets for treatment.

Baltu and Baltu illustrated how such injection principles can be extended to congenital unilateral lower-lip palsy. In EMG-proven weakness of the DAO, DLI, and platysma muscles, symmetry can be restored by selectively weakening the intact side with relatively small, well-placed BoNT-A injections. Their 18-year-old patient received onabotulinumtoxinA injections into the contralateral DAO muscle and along right platysmal bands, resulting in the correction of lower-lip depression during smiling and grimacing without rest asymmetry or functional compromise over three years of 6-monthly treatments [32].

Carrè et al. described a detailed technical plan and injection strategy. A pre-injection assessment is essential for understanding the history and evolution of the paralysis. Muscle tone is analyzed globally at rest and then for each muscle individually at maximum contraction during a standardized battery of movements: eyebrow elevation, full palpebral closure (with and without forcing), nose wrinkling, smiling, labial protrusion, pouting, whistling, and lowering of the lower lip [33]. To rate the synkinesis burden, dedicated scales such as the Sunnybrook Facial Grading System (SB-FGS) and the Synkinesis Assessment Questionnaire are employed. An analysis of wrinkles and residual hypotonia leads to a diagrammed and tailored injection plan, with precise, localized dosages of BoNT-A across all facial thirds to balance asymmetry and relieve functional deficits [33].

Cabin and colleagues emphasized the importance of using a standardized sequence of movements to document the patient’s disease and improvements: neutral face, small smile, big smile/grin, raised eyebrows, furrowed eyebrows, puckered mouth/whistling, lower lip down, and upper lip up. They also recommended using a grading system for synkinesis (such as the SB scale) at each visit. The recommended injection protocol includes targeting the orbicularis oculi to reduce eyelid aperture narrowing and the platysma to reduce neck banding and tightness. Additionally, they focused on the buccinator and the DAO to improve oral commissure excursion and the smile mechanism. Other targets included the mentalis, frontalis, and corrugator supercilii. Selective neuromodulation on the unaffected side was used to create resting and dynamic symmetry and to modulate hyperkinesis. Specifically, the frontalis and orbicularis oculi were targeted to match the contralateral rhytid (wrinkle) patterns. Other targets included LLSAN to reduce visibility of the upper teeth and DLI to reduce visibility of the lower teeth. According to this strategy, initial low doses (below standard) are used, followed by a 2-week follow-up to assess the need for additional injections. Furthermore, they advocated for systematically coupling injections with neuromuscular retraining and mirror biofeedback [12]. This combined protocol is described as synergistic, enhancing the toxin’s effects and producing improvements that persist even after the pharmacologic effects of the BoNT-A have dissipated, a finding consistent with, though not directly demonstrated by, the longitudinal photographic improvement observed in our Case 1.

De Sanctis Pecora and Shitara extended the same concepts with an algorithm that starts with a standardized asymmetry and functional assessment using nine front views (rest, brow elevation, complete eye closure, nose wrinkling, grin, pucker, whistle, pout, and lower-lip depression) plus a profile view to assess the action of the platysma specifically [34]. This approach was combined with the consistent use of the House–Brackmann (HB) or SB-FGS to determine severity. Additionally, the Synkinesis Assessment Questionnaire was used as a clinical tool to quantify the burden of synkinesis and inform treatment decisions. The analysis was then translated into a muscle-by-muscle injection map on the nonparalyzed side, specifying both indications and technique. Finally, they recommended treating platysma and lower-face depressors aggressively but with incremental dosing, starting with low initial doses and incrementing every 2 weeks to define an individualized dose map that maximizes symmetry and smile excursion without causing a “freezing” expression [34].

In a previous publication, we provided our experience-dictated values [31]. We now present an updated table, expanding on the previously cited studies [12,33,34] (Table 1). These values should be understood as practical guidance rather than evidence-based standards.

## 5. Use of Functional Scales

Numerous assessment tools are used in scientific studies. However, time constraints are a common limitation in outpatient practice. In clinical practice, the assessment of facial paralysis primarily relies on standardized clinician-led grading systems, with the House–Brackmann (HB) [35] and Sunnybrook (SB) [36] scales being the most frequent, in addition to overall satisfaction with treatment in daily life, which is always inquired, with a rating from 0 to 10 [34,37].

The HB scale is a six-point grading system that categorizes patients into general functional groups. Clinicians evaluate the face based on gross appearance, symmetry at rest, and motion in specific areas, such as the forehead, eyes, and mouth. Grading categories of dysfunction include grade I (normal); grade II (mild) with light weakness, normal symmetry at rest, complete eye closure with minimum effort; grade III (moderate), with obvious but not disfiguring asymmetry, noticeable synkinesis, and complete eye closure with effort; grade IV (moderately severe), with obvious weakness or disfiguring asymmetry, no forehead movement, and incomplete eye closure; grade V (severe), with only barely perceptible motion and asymmetry at rest; and grade VI (total paralysis), with no movement at all [35].

The SB-FGS is a more detailed tool that provides a quantitative composite score ranging from 0 to 100. It assesses three distinct dimensions: resting symmetry, voluntary movement excursion, and synkinesis. Resting symmetry evaluates the eye, nasolabial fold, and corner of the mouth on a point scale; voluntary movement is scored on five standard expressions (forehead wrinkle, gentle eye closure, open-mouth smile, snarl, and lip pucker) each graded from 1 to 5; synkinesis is scored as the degree of involuntary muscle contraction associated with the five expressions, which is graded from 0 to 3; the final composite score is calculated by weighting these dimensions (e.g., voluntary movement is multiplied by 4, resting symmetry by 5) and subtracting the resting and synkinesis totals from the voluntary movement total [36].

In our clinical practice, we use the SB-FGS because its higher sensitivity to clinical changes enables better monitoring of recovery, a separate evaluation of different facial regions, and more thorough accounting for the complexity of synkinesis.

In Choi et al., functional scales such as the SB-FGS were central to both patient selection and outcome evaluation, underscoring the role of standardized instruments in guiding BoNT-A treatment for facial palsy. All patients with long-standing post-paralytic synkinesis and contralateral hypertrophy were required to have an SB synkinesis subscore greater than 5 and a composite “dynamic facial asymmetry ratio” (developed by the authors to grade improvements after treatment) below 0.9 at baseline. Subsequent improvements in synkinesis, symmetry, and overall facial function were quantified as a change in the total SB score, alongside a significant rise in the developed dynamic facial asymmetry ratio [38].

Similarly, De Sanctis Pecora and Shitara argued that objective grading tools are indispensable for planning and monitoring BoNT-A treatment, recommending the selection of a single facial nerve grading system and its consistent application over time [34].

Lee and co-workers further highlighted the value of the SB scale as a measure of the long-lasting effects of BoNT-A treatment. The positive effects of BoNT-A in repeated injection sessions, together with a structured rehabilitation program using half-mirror biofeedback exercises, were monitored using the SB scale to track improvements. In their patient series, the SB score consistently increased after each treatment session, documenting not only short-term chemodenervation effects but also a durable, functionally meaningful improvement in symmetry and synkinesis over two years of combined toxin and exercise therapy [39].

Müller and co-workers employed AI-based tools to provide objective measurements of facial symmetry, thereby reducing the interobserver variability common in manual assessments. In their prospective observational study, a series of patients with non-flaccid facial palsy who underwent BoNT-A treatment were analyzed using Emotrics^®^ (Massachusetts Eye and Ear Infirmary, Boston, MA, USA), an AI-based tool for static objective assessments, FaceReader^TM^ (version 8.1; Noldus Information Technology, Wageningen, The Netherlands), an AI-driven software tool that analyzes spontaneous and evoked emotions such as joy, sadness, anger, surprise, disgust, fear, and neutrality, and more established PROMs such as the FaCE questionnaire and the Face Disability Index (FDI). According to these assessments, BoNT-A significantly improved objective symmetry and social/aesthetic self-ratings while leaving functional FDI scores and automated emotion recognition largely unchanged. The authors suggested that this may be the result of an objectively improved symmetry, but accompanied by the reduced activation of dynamic muscles [37].

Although the HB and SB-FGS scales are the most frequently adopted instruments in clinical practice, both have their limitations. The HB scale is simple and easy to use. Still, it cannot detect small yet clinically meaningful differences in facial function, does not separately assess synkinesis as a distinct dimension, and cannot evaluate individual facial thirds. This is especially relevant when monitoring the targeted, region-specific effects of BoNT-A treatment [35,40]. The SB-FGS multidimensional composite scoring structure addresses some of these limitations and has demonstrated superior sensitivity to clinical change [36,39]; however, its interobserver reliability is only moderate, with the synkinesis subscore (the most directly relevant dimension to BoNT-A treatment monitoring) showing the greatest interrater variability [40]. Neither scale includes patient-reported measures, so improvements in quality of life, social functioning, and emotional well-being are not captured. Complementary instruments, such as the FaCE questionnaire for broader functional and psychosocial assessment, are therefore useful [16,37]. From a practical point of view, the HB scale is appropriate for rapid initial grading in general outpatient and emergency settings. At the same time, the SB-FGS is best suited to dedicated facial nerve clinics where standardized assessment is a routine component of the clinical workflow. AI-based objective tools such as Emotrics^®^ and FaceReader™ offer the advantage of eliminating interobserver subjectivity, but require specialized software and imaging instruments [37].

## 6. Use of Instrumental Guidance

In complex cases of synkinesis with significant aberrant reinnervation, an approach that combines BoNT-A treatment with muscle localization and functional assessment techniques may help optimize injection accuracy. In recent years, the use of ultrasound (US) and EMG has increased [41,42,43,44,45]. Although anatomical landmark-guided injections can have satisfactory results in expert hands, instrumental guides enable more precise localization for toxin administration and appropriate dosage selection. US provides morphological and topographical visualization of subcutaneous structures, whereas EMG directly identifies the active muscle portions that contract abnormally. This is made possible by needles specifically designed to record muscle signals and to enable simultaneous injection in the selected areas. This approach can help optimize dosing, minimize side effects, and improve overall outcomes, ensuring greater stability of therapeutic effects over time [42].

Supplementary Video S2 (https://doi.org/10.5281/zenodo.19094685, accessed 18 May 2026) shows a representative patient undergoing EMG-guided BoNT-A injection in our clinic. The patient had an outcome of post-traumatic acute total right facial paralysis with a petrous bone fracture. The paralysis was graded 6 on the HB scale. Motor recovery began 5 months post paralysis onset with synkinesis. The treatment started 12 months after paralysis, in parallel with a motor re-education program. The SB score improved from 52 (pre-treatment) to 67, with an injection treatment plan that followed a 4–5 month schedule. The therapeutic effect was appreciated by the patient, especially in facial symmetry and synkinesis of the lower third of the face.

A recent comprehensive review of the available localization techniques is provided by Karp and co-workers. It compares manual, EMG, electrical stimulation, US, and combined approaches in terms of precision and outcomes. Manual placement, which relies on tactile sensitivity and medical experience, may be adequate for large superficial muscles. However, approaches that rely on instrumental guidance significantly improve injection accuracy, particularly for small or deep muscles. EMG is most valuable for measuring muscle activity and hyperactivity, which is especially relevant in the context of facial paralysis. US, on the other hand, provides the real-time visualization of anatomic structures, which is important for ensuring safe and targeted delivery. Combined modalities, such as EMG and US, are particularly advantageous when both functional and anatomic guidance are required. In the context of facial paralysis management, these findings reinforce the clinical relevance of technology-assisted localization to optimize selective chemodenervation, enhance symmetry, and minimize unwanted diffusion-related weakness [42].

EMG can be used to systematize localization and dose titration across repeated BoNT-A injections [43]. Homma and colleagues used integrated EMG (iEMG) to quantify the activity ratio between the affected and healthy sides of the face in patients with synkinesis following facial palsy. The calculated symmetry index between before treatment and at least 26–32 weeks after the first, second, and third treatment showed sustained, dose-independent improvements in synkinetic activation patterns, particularly for the orbicularis oculi during smiling and lip puckering, and for the platysma during eye closure and lip puckering. However, only modest changes in SB synkinesis subscores were recorded, highlighting how instrumental recording can provide the most sensitive feedback to more precisely adjust dosages across multiple treatment sessions [43].

Expanding from EMG to US, the latter can provide a non-invasive method for both visualizing the facial nerve and guiding intramuscular BoNT-A injections, allowing the toxin to be deposited under real-time guidance into the intended target while avoiding nearby structures that, in landmark-guided injections, are responsible for complications such as ptosis, diplopia, lagophthalmos, dysphonia, or unwanted paralysis [44].

The efficacy of US-guided injections has been further confirmed on cadaver models. Typical synkinetic muscles, such as the orbicularis oculi, DAO, and mentalis, as well as the lacrimal gland, were injected with ink to mimic BoNT-A injections, either under US or landmark guidance. The injection accuracy was then evaluated, and the results showed that US guidance improved both localization and dose delivery compared with landmark techniques. The increased precision can reduce common clinical complications such as ptosis or double vision [45].

The choice of guidance modality in real-world clinical settings relies on practical considerations. Landmark-guided injections require no specific equipment, but do require appropriate experience of the performing clinicians. Nevertheless, they are associated with greater variability in injection accuracy for small or deep muscles. EMG guidance provides valuable functional information on target muscle activity and enables better dose titration, but it requires dedicated equipment, disposable EMG injection needles, and specific training. Ultrasound guidance provides real-time anatomical visualization and reduces the risk of complications from accidental injection into adjacent structures, but also requires appropriate equipment and training. Rigorous training, standardized injection maps, and systematic retouch sessions are a clinically valid alternative in settings where instrumental guidance is unavailable due to cost or infrastructure limitations. Instrumental guidance can be reserved for complex cases in settings where it is available.

## 7. Functional Microsurgery and Botulinum Toxin: Hand in Hand

Reconstructive microsurgical techniques have become increasingly refined to restore facial movement in paralysis and improve aesthetic outcomes by using donor nerves, including the contralateral facial nerve, the motor branch of the trigeminal nerve, the spinal accessory nerve, and the hypoglossal nerve. In cases of both paralysis and aberrant recovery with synkinesis, these techniques aim to achieve effective reinnervation [46,47,48,49]. In this context, BoNT-A plays a fundamental role, as it can be used either as an initial therapeutic intervention or to simulate surgical outcomes, thereby providing patients with an understanding of the sensation of reduced synkinesis. BoNT-A treatment has the advantage of being reversible, unlike surgery, which is permanent, helping patients realize the potential outcomes of functional surgery and decide whether to continue medical treatment or proceed with surgery [12,27,50,51].

BoNT-A has also been shown to significantly improve outcomes in patients undergoing cross-facial nerve grafting with secondary microcoaptations to treat post-facial paralysis synkinesis. BoNT-A reduces aberrant co-contractions, thereby optimizing the environment for reinnervated muscles [52].

Furthermore, after nerve graft procedures, even when facial motor function has recovered, patients often report uncoordinated movements that may occur during fiber regrowth, causing functional and aesthetic discomfort. BoNT-A can help refine and correct such movements [12].

The study by Ma and co-workers evaluated the efficacy of BoNT-A as a follow-up treatment after reconstructive nerve surgery in patients with severe oral commissure drooping due to facial paralysis following acoustic neuroma resection. BoNT-A was injected in low doses into the LLS, LLSAN, major zygomatic muscle, minor zygomatic muscle, musculus risorius, and DLI of the unaffected side, significantly improving facial symmetry and the long-term aesthetic appearance by altering motor recovery and brain plasticity. The findings suggest that relaxing active muscles creates a “window of opportunity”, allowing the brain to adapt and maintain better facial alignment [18].

## 8. Conclusions

BoNT-A is a clinically valuable tool in the management of facial paralysis, which offers targeted chemodenervation with the potential to reduce static and dynamic asymmetry, alleviate synkinesis and hyperkinesis, and improve functional and psychosocial outcomes across the acute and chronic phases. The three presented cases illustrate BoNT-A’s versatility across different clinical scenarios, with improvements in functional grading scores, as also documented through rigorous photography. This approach not only bridges aesthetic symmetry and motor recovery but has also been hypothesized to create a therapeutic window for neuroplasticity. This was not directly tested in the cases presented here and remains speculative, but still supports its role as a valuable adjunct to micro-surgery and rehabilitation to optimize long-term patient satisfaction, to be considered early on after paralysis onset as a therapeutic tool that affects function and aesthetics.

## Figures and Tables

**Table 1:** Summary of BoNT-A dosing and injection plans to treat facial paralysis patients.

Muscle (Group)		Dose Range (Units per Session) *	Injection Level	Injection Depth (mm)
**Affected side**				
	Procerus	4–8	Intramuscular	6–8
	Corrugator supercilii	4–10	Intramuscular	6–8 (origin)/2 (insertion)
	Depressor supercilii	1–2	Subdermal	Superficial
	Frontalis ^#^	1.5–18	Intramuscular or Intracutaneous	2–4
	Orbicularis oculi	2.5–10	Intracutaneous	2–3
	Orbicularis oculi (endopalpebral portion)	2–4	Intracutaneous (very superficial)	1
	Lacrimal gland	1–20	Intraglandular	Transconjunctival
	Nasalis (transverse portion)	2–4	Intramuscular	3–4
	Zygomaticus major	1–5	Intramuscular (origin of the muscle)	8–10
	Zygomaticus minor	2	Superficial intramuscular	5
	Risorius	1–3	Intramuscular	4
	LLSAN	2–7.5	Intramuscular	4–8
	LLS	1–2	Intramuscular	4–8
	Buccinator	2–20	Endobuccal route	Perpendicular to the mucosa
	Orbicularis oris	1–6	Superficial/Intradermal	1–2
	DAO	0.5–10	Intramuscular (deep at bony origin)	1–3 points along the DAO line
	DLI	1–3	Intramuscular	2–3
	Platysma (lateral and anterior)	10–60	Intracutaneous/Superficial intramuscular	1–2
	Mentalis	2.5–10	Deep intramuscular	6–10
	SCM	5–15	Intramuscular	Deep
	Masseter	5	Intramuscular	Deep
**Unaffected side**				
	Corrugator supercilii	2–4	Intramuscular	6–8 (origin)/2 (insertion)
	Frontalis	2–10	Intramuscular or Intracutaneous	2–4
	Orbicularis oculi	4–10	Intracutaneous	2–3
	Orbicularis oculi (endopalpebral portion)	2–4	Intracutaneous	1
	Nasalis	2	Intramuscular	3–4
	LLSAN	2.5–7.5	Intramuscular	4–8
	DLI	2.5–5	Intramuscular	2–3
	Platysma (lateral and anterior)	20–34	Intracutaneous	1–2
	Mentalis	3–5	Intramuscular	6–10 (origin)/2 (insertion)
	Masseter	5	Intramuscular	Deep

^#^ Note: Frontalis dosing on the affected side varies significantly depending on whether the patient presents with flaccid hypotonia (where higher doses balance the healthy side) or paradoxical hyperkinesis (Babinski’s sign). Data extracted from [12,31,33,34]. *, Units from [12,31] refer to onabotulinumtoxinA; units from [33] refer to International Units, which are equivalent to onabotulinumtoxinA; units from [34] refer to incobotulinumtoxinA, which can be converted to onabotulinumtoxinA with a 1:1 conversion ratio. Therefore, all presented units are equivalent and comparable to onabotulinumtoxinA. Abbreviations: DAO, depressor anguli oris; DLI, depressor labii inferioris; LLS, levator labii superioris; LLSAN, levator labii superioris alaeque nasi; SCM, sternocleidomastoid.

## Data Availability

The data associated with the clinical cases are not publicly available as they contain information that could compromise the privacy of the participants, but are available from the corresponding author upon reasonable request.

## Supplementary Materials

The following supporting information can be found at: https://doi.org/10.5281/zenodo.19331961, accessed on 18 May 2026, Video S1: Smile in a woman with facial paralysis; https://doi.org/10.5281/zenodo.19094685, accessed on 18 May 2026, Video S2: EMG-guided BoNT-A injection for the treatment of facial paralysis.

## Abbreviations

The following abbreviations are used in this manuscript:

BoNT-A	Botulinum neurotoxin serotype A
DLI	Depressor labii inferioris
DOA	Depressor anguli oris
EMG	Electromyography
GAIS	Global Aesthetic Improvement Scale
HB	House-Brackmann (scale)
LLS	Levator labii superioris
LLSAN	Levator labii superioris alaeque nasi
SB-FGS	Sunnybrook Facial Grading System
U	Units
US	Ultrasound
